# Factors Affecting Recovery from Post-Traumatic Amnesia During Inpatient Brain Injury Rehabilitation: A Retrospective Cohort Study

**DOI:** 10.3390/life16020203

**Published:** 2026-01-26

**Authors:** Rathi Ratha Krishnan, Yuhan Yang, Emily Yee, Karen Sui Geok Chua

**Affiliations:** 1 Institute of Rehabilitation Excellence (IREx), Tan Tock Seng Hospital, 11 Jalan Tan Tock Seng, Singapore 308433, Singapore; emily.yee@nhghealth.com.sg (E.Y.); karen.chua@nhghealth.com.sg (K.S.G.C.); 2 Lee Kong Chian School of Medicine, Nanyang Technological University, Singapore 639798, Singapore; yang.yuhan@mohh.com.sg; 3 Yong Loo Lin School of Medicine, National University of Singapore, Singapore 541397, Singapore; 4 MOH Holdings Pte Ltd., Singapore 139691, Singapore; 5 Clinical Research and Innovation Office (CRIO), Tan Tock Seng Hospital, Singapore 308433, Singapore; 6 Rehabilitation Research Institute of Singapore (RRIS), Nanyang Technological University, Singapore 639798, Singapore

**Keywords:** post-traumatic amnesia, traumatic brain injury, rehabilitation, inpatient, functional independence measure, geriatric, Westmead Post Traumatic Amnesia Scale

## Abstract

Background: Longer post-traumatic amnesia (PTA) durations in traumatic brain injury (TBI) are associated with worse functional outcomes, poorer cognition, and persistent disability. A retrospective cohort study was conducted to evaluate factors affecting PTA duration and emergence. Methods: Data extraction of discharged records of adult TBI was performed between 1 April 2022 and 4 May 2023. Independent variables collected include socio-demographic, acute TBI, and rehabilitation characteristics. Admission/discharge Functional Independence Measure (FIM) was the main rehabilitation outcome measure charted. Dependent variables included PTA duration ≤ 30 days, >30 days, and PTA emergence. Results: A total of 189 datasets were analysed. Median age (IQR) 64 years (26), 145 males (76.7%), and 64.6% >55 years. PTA ≥ 30 days were correlated with the following factors: older age (66 years vs. 59.5 years, *p* = 0.017), presence of ICU admission (75.2% vs. 61.4%, *p* = 0.029), longer ICU stays (5 days vs. 3 days, *p* = 0.001), and longer duration of inpatient hospitalization (acute length of stay, ALOS 23 days vs. ALOS 14 days, *p* < 0.001). Age ≥ 55 years were 5.6 times as likely (*p* = 0.011) to be in prolonged PTA, an additional day’s stay in the acute hospital increased the odds by 1.15 (*p* < 0.001), and every score lost in the total admission FIM from 40 and below increased the odds of prolonged PTA by 3.35 times (*p* = 0.014). Conclusions: This study demonstrated that older age at TBI onset and longer ALOS significantly increased the risk of prolonged PTA duration. Conversely, higher admission FIM score, lower age at admission, and shorter ALOS were associated with lower PTA duration.

## 1. Introduction

Across all ages, traumatic brain injury (TBI) constitutes a substantial contributor to disability and death [1]. More than 50 million TBIs take place annually across the globe, with falls exceeding road accidents as a major cause due to an ageing population [2,3]. TBI survivors are at greater risk of developing cognitive impairment at a younger age. Long-term mortality risk is also increased [4,5].

Post-traumatic amnesia (PTA) is a subtype of delirium which can affect patients with moderate to severe TBI as they emerge from the acute or post-coma phase. It can consist of a spectrum of behavioural disturbances, including post-traumatic confusion, impaired attention, memory, cognitive deficits, post-traumatic agitation, and aggression. Physiological changes like sleep disturbances, impaired balance, reduced visual perception, and visual motor performance also occur during this period [6]. Many scales have been used to measure and track PTA duration (days), progress, and emergence, for example, the Westmead Post Traumatic Amnesia Scale (WP-TAS), abbreviated WP-TAS and Orientation Log (O-Log) [7,8,9].

Several authors have alluded to the important role of PTA duration in prognosticating various outcomes in the acute and long-term recovery trajectory of TBI [10]. For example, longer PTA durations are associated with worse functional outcomes, poorer cognition, and increased chances of persistent disability [6,11]. Walker et al. reported that in cases of moderate to severe TBI, achieving emergence from PTA within four weeks is associated with a decreased likelihood of severe disability, indicating a higher probability of good recovery at the two-year mark. In contrast, if the duration of PTA exceeds eight weeks, the prospects for good recovery diminish significantly [12].

Factors affecting PTA duration are multifactorial. They can range from injury severity, location of brain lesion, age and cognitive status of the patient, and post-injury complications and treatments. In a study by Sherer et al., age, years of education, Glasgow Coma Scale (GCS), length of coma, pupillary responsiveness, and intracranial operations were predictive of PTA duration [13].

In older adults, evaluating and managing PTA is challenging due to factors such as pre-existing cognitive impairment, multiple comorbidities, and age-related brain atrophy, all of which can complicate assessment and prognosis. They are more likely to experience prolonged PTA and poorer functional outcomes, with age serving as a consistent predictor of negative functional outcome after moderate-to-severe TBI [14,15].

The aims of this study were to study the PTA recovery, i.e., duration of PTA (days) and emergence at the time of discharge during inpatient acute brain injury rehabilitation, using standardized PTA administration scales and elucidate pertinent demographic, injury, acute and rehabilitation factors affecting these outcomes.

## 2. Materials and Methods

### 2.1. Study Design and Setting

A retrospective cohort study of acute adult TBI inpatients’ electronic medical records (EMRs) was conducted from 1 April 2022 to 4 May 2023. All inpatients originated from a single rehabilitation unit, the Tan Tock Seng Hospital (TTSH) Rehabilitation Centre, Singapore, which has direct links to a national level neurotrauma centre, the National Neuroscience Institute, Singapore. All patients were pre-screened via staff rounds by physiatrists prior to admission.

### 2.2. Study Population

All patients had previously completed inpatient rehabilitation at TTSH Rehabilitation Centre, Singapore. The study inclusion criteria were patients above 21 years old with first-ever TBI confirmed by neuroimaging and neurosurgeons, admitted for the main purpose of rehabilitation within 3 months of TBI onset. Patients who admitted for non-rehabilitation reasons and those with incomplete PTA data, admission, or discharge Functional Independence Measure (FIM) data were excluded [16,17].

### 2.3. Description of Inpatient Rehabilitation Programme

Located in central-north Singapore, the Tan Tock Seng Hospital (TTSH) Rehabilitation Centre is a 121-bed tertiary inpatient facility. It operates in close integration with a Level II trauma and national neurotrauma centre, collectively addressing the acute and chronic healthcare requirements of approximately 1.5 million residents. Both institutions utilize a unified electronic medical record (EMR) system, facilitating seamless transitions of care.

Admission and Consultation Framework:

Potential transfers to the specialized Brain Injury Rehabilitation Unit undergo rigorous screening via physiatry consultations or specialist-led rounds to confirm medical stability and rehabilitation indications. For patients with traumatic brain injury (TBI) remaining in acute neurosurgical wards, multidisciplinary consultations are initiated early to streamline the recovery trajectory.

Unit Staffing and Resource Allocation:

During the study period, the TBI unit maintained an 18-bed capacity. Staffing ratios for nursing and therapy were maintained at 1:7–8 patients. The interdisciplinary team comprised Physiotherapists and Occupational Therapists, Speech and Language Pathologists, Psychologists and Medical Social Workers, Dietitians (weekly), and Orthotists (as clinically indicated).

The Rehabilitation Program:

Managed by a consultant rehabilitation physician, the program mandates three hours of interdisciplinary therapy daily, five and a half days per week. Clinical management is structured around collaborative goal setting and weekly multidisciplinary reviews. Discharge planning—incorporating estimated dates of transition—is dictated by the achievement of functional goals, family caregiving capacity, and the intended discharge destination.

Core therapeutic interventions include the following:

Neuromodulation: Reality orientation, sleep–wake cycle regulation, and targeted neuropharmacology.

Behavioural and Functional Training: Agitation management and Basic Activities of Daily Living (ADL) coaching.

Cognitive Monitoring: Post-traumatic amnesia (PTA) charting is initiated once patients demonstrate adequate arousal, attention, and linguistic capacity without prohibitive agitation. Notably, emergence from PTA is not a prerequisite for discharge from the inpatient program.

The main rehabilitation outcome measure used was the Functional Independence Measure^®^ [16,17] (18 minimum score, 126 maximum score), measured within 72 h of rehabilitation admission (Ta-FIM) and planned and anticipated rehabilitation discharge (Td-FIM). Hence, FIM ≥ 91 indicated modified-to-independent levels, implying 6–7 points each, aggregated across the 18 domains.

The duration of post-traumatic amnesia (PTA) was defined as time (days) from TBI onset date till the return of continuous memory of ongoing events. PTA duration was measured by rehabilitation therapists using either the Westmead PTA scale (WPTAS) [18,19,20] or Orientation Log (O-Log) [8,21] in the following manner. For patients < 80 years of age without prior causes of cognitive impairment (mild cognitive impairment, dementia, stroke, neurodegenerative diseases), the WPTAS was used to assess PTA duration in days. WPTAS consists of 7 orientation items and 5 memory assessing prospective memory items. The scale is administered daily, and PTA emergence levels are achieved on the first of 3 days upon obtaining a score of 12/12 or until the day of discharge from inpatient rehabilitation if 12/12 is not achieved by then. At the first 12/12 score, the 3 memory picture cards are changed. The duration of PTA was determined in days, calculated from the date of TBI till the first of the 3 consecutive daily scores of 12/12, denoting PTA emergence, or until the day of hospital discharge, if not emerged. If the patient failed to achieve criterion score on either WMPTAS or O-Log by discharge, patients were deemed PTA-non-emerged. For durations of >28 days of PTA, the first day of a 12/12 score was taken as the date of emergence [18].

The O-Log, rather than WMPTAS, was used if patients were >80 years of age or had prior stroke or dementia or known cognitive impairment. Two consecutive scores of >24/30 on the O-Log met criterion for emergence from PTA [8]. PTA duration was taken as total hospital length of stay if patient failed to attain emergence on either of the 2 scales. [19]. We also determined prolonged PTA duration as those exceeding 30 days.

### 2.4. Data Collection Process

Data extraction from EPIC/CPSS was performed, and a case record form was constructed. Data was subsequently deidentified prior to analysis. Anonymized data from 2 previous studies (NHG DSRB 2020/00298 and 2022/00168) were combined with the current dataset to form the final dataset.

Independent variables collected include socio-demographic characteristics (age, gender, ethnicity, premorbid employment status, and presence of comorbidities). The Charlson Comorbidity Index (CCI), an estimation of risk of death from comorbidities, was calculated for each dataset [22].

Acute TBI characteristics collected were aetiology of TBI, admission Glasgow Coma Scale (GCS), presence of ICU admission, ICU length of stay (LOS), presence of neurological intervention (extra ventricular drainage, intracranial pressure monitor, craniotomy, decompressive craniectomy, clot evacuation, tracheostomy, ventriculoperitoneal shunt), presence of skull fracture, presence of acute TBI seizures, and acute [8]. TBI acute severity was further classified based on admission GCS into the following 3 categories: mild (GCS: 13–15), moderate (GCS: 9–12), or severe (GCS: 3–8) [23,24].

Rehabilitation characteristics extracted include the rehabilitation LOS, PTA duration (days), and PTA emergence by criteria as previously specified; presence of medical and neurosurgical complications during rehabilitation; and presence of acute unplanned returns (ACUR) to acute wards due to neurosurgical or medical causes. For ACUR patients, rehabilitation LOS was based on number of days spent in the rehabilitation centre.

The admission and discharge FIM were the main outcome measures charted. FIM is a valid measure of functional dependency and degree of assistance for patients with TBI [25]. Total admission FIM (Ta-FIM), total discharge FIM (Td-FIM), and their cognitive and motor components were recorded upon rehabilitation admission and discharge [26]. Functional independence level was determined as Td-FIM ≥ 91.

Discharge characteristics including discharge disposition (home or institutional care) and the need for a caregiver were extracted.

### 2.5. Statistical Analysis

Statistical analysis was performed using R Studio (Version 4.2.0). Descriptive statistics were calculated for all variables, with continuous variables presented using median and interquartile range (IQR) due to non-normal distribution, while categorical variables are presented using frequencies and percentages.

Univariate analysis was conducted for characteristic comparison between patients with PTA duration of ≤30 days and >30 days as well as between PTA emergence and PTA non-emergence groups. For continuous variables, the Mann–Whitney U test was later utilized to examine differences between groups. For categorical variables, Pearson’s Chi-Square test was utilized to identify associations.

Variables that showed statistical significance (*p* < 0.05) in univariate analysis were then considered for inclusion in the multivariate models. Also, binary logistic regression was selected to identify independent predictors of prolonged PTA duration (>30 days) as well as PTA emergence. The regression models were built using a stepwise approach, with the dataset randomly split into training (70%) and testing (30%) sets for model validation.

Model performance was later assessed using accuracy measures, and results were presented as odds ratios (OR) with 95% confidence intervals (CI). Statistical significance was set at *p* < 0.05 for all analyses. Missing data was omitted during the model building process, with sample size for each analysis clearly stated where data was incomplete.

Multicollinearity among candidate predictors was assessed using the variance inflation factor (VIF). No evidence of problematic multicollinearity was observed, as all candidate predictors had VIF values < 5.

The level of all statistical tests was *p* < 0.05.

Guidelines from STROBE (www.strobe-statement.org accessed on 9 November 2025) were followed to strengthen the reporting of this study (refer Supplementary Materials S2; STROBE checklist).

## 3. Results

Altogether, 267 EMR datasets were screened, and a total of 78 were excluded due to missing FIM/PTA data (53) and presence of previous TBI (25). Thus, 189 datasets were analysed, categorised as 88 with shorter PTA (≤30 days) and 101 with >30 days. Overall, the median age (IQR) was 64 years (26) with 145 males (76.7%), 122 (64.6%) aged >55 years, and falls accounted for 55% of TBI

Table 1 shows the univariate analysis of independent factors between PTA duration ≤ 30 days and >30 days.

In terms of demographic and TBI characteristics, longer durations of PTA > 30 days were correlated with the following factors: older age (66 years vs. 59.5 years, *p* = 0.017), presence of ICU admission (75.2% vs. 61.4%, *p* = 0.029), longer ICU stays (5 days vs. 3 days, *p* = 0.001), and longer duration of inpatient hospitalization (ALOS 23 days vs. ALOS 14 days, *p* < 0.001 and RLOS 34 days vs. RLOS 21 days, *p* < 0.001).

Sex, race, admission GCS, neurological interventions, seizures, and complications during rehab stay were not significantly correlated with PTA duration.

In terms of rehabilitation outcome, those with longer PTA durations had significantly lower admission and discharge FIM (total admission FIM 48 vs. 68, *p* < 0.001, total discharge FIM 83 vs. 102.5, *p* < 0.001). They also had a lower proportion of PTA emergence (76.2 vs. 87.5%, *p* = 0.047) and a higher proportion who were institutionalized post-discharge (26.7% vs. 6.8%, *p* < 0.001).

Table 2 shows variables predicting PTA duration of more than 30 days using the binary logistic regression model. Age of 55 years and upward were 5.6 times as likely (*p* = 0.011) to be in prolonged PTA, an additional day’s stay in the acute hospital increased the odds by 1.15 (*p* < 0.001), and every score lost in the total admission FIM from 40 and below increased the odds by 3.35 times (*p* = 0.014).

Table 3 compares variables affecting PTA emergence using the binary logistic regression model. The following were statistically significant as negative predictors for PTA emergence: increasing PTA duration (OR 0.93, *p* < 0.001) and age ≥ 55 (OR 0.161, *p* = 0.031). Increasing days of ALOS and admission cognitive FIM scores were statistically significant positive predictors for PTA emergence (OR 1.116, *p* = 0.012 and OR 1.151, *p* = 0.007, respectively).

## 4. Discussion

### 4.1. Socio-Demographic and Acute TBI Characteristics Affecting PTA Duration

The World Health Organization (WHO) generally defines elderly as those aged 60 years and above [27], while the Centers for Disease Control and Prevention (CDC) uses 65 years as the age cutoff when reporting health indicators in the elderly [28]. However, in our study, age was dichotomised at 55 years. According to a paper by Weihs et al. (2020), age > 55 years was a significant risk factor in predicting late-phase mortality in polytrauma patients with severe TBI using ROC analysis [29]. PTA duration of more than 30 days as the cutoff for prolonged PTA was chosen as it is the local institution practice.

This study demonstrated that older age (66.0 years vs. 59.5 years) was correlated with a longer duration of PTA > 30 days. Additionally, those aged 55 years and upward were 5.6 times more likely to be in prolonged PTA. This concurs with a large-scale study by Hukkelhoven et al. (2003) [30]. Analysing a cohort of 5600 patients with severe TBI, the authors stratified outcome data by age and reported substantially worse prognosis in the older group. Specifically, patients aged >55 years demonstrated a 52% mortality rate and 74% rate of unfavourable outcome at 6 months post-injury [30].

Our results are consistent with earlier cohort studies showing that older adults tend to have poorer outcomes after TBI. Several large analyses have reported a steady rise in mortality and severe disability with each decade of age [14,15,31,32]. This pattern matches what we observed: older patients in our cohort were more likely to remain in PTA for more than 30 days and had lower functional scores at admission. These poorer outcomes may reflect age-related health issues, pre-existing comorbidities, and differences in injury severity, all of which are more common in older individuals [14,15]. Some studies have noted that older adults with mild TBI may recover at rates similar to younger patients. However, the overall evidence still shows that advanced age is linked to higher mortality and more severe disability, especially in moderate-to-severe TBI [14,33,34]. Frailty, which is more common in this age group, likely adds to this risk.

Several factors may explain why older adults stayed in PTA longer in our study. Ageing is associated with reduced neuroplasticity and lower cognitive reserve, which can slow recovery after brain injury [35,36]. Older patients also tend to have more medical complications, which may disrupt early rehabilitation and further delay emergence from PTA.

Degeneration of white-matter pathways and a higher burden of chronic disease in older adults may also make recovery slower [37,38,39]. Cho et al. [38] were able to demonstrate white matter integrity post-TBI using Diffusion tension imaging (DTI). Their study showed that injury severity to eight neural structures involved in cognitive function was relative to the duration of PTA. Reduced physiological reserve and more frequent medical issues during hospitalization can further add to delays to recovery [14,40].

This is reflected in the patients of our study. Those who were in prolonged PTA > 30 days demonstrated a statistically significant lower admission FIM cognitive score (16 vs. 21, *p* = 0.001). This suggests that in our study, the patients who fared worse had reduced cognitive reserve.

### 4.2. PTA Duration and Complications

Patients with a PTA duration > 30 days had longer ICU stays (5 days vs. 3 days) and longer duration of inpatient hospitalization (ALOS 23 days vs. 14 days and RLOS 34 days vs. 21 days). Prolonged PTA is by itself a risk factor for increased hospitalization length [41]. A local study by Chua et al. (2025) reported that TBI patients with prolonged PTA durations were 4 times more likely to have prolonged RLOS > 30 days, while every day in PTA prolonged 0.2 days of RLOS [42].

In this study, an additional day’s stay in the acute hospital increased the odds of prolonged PTA by 1.15. This might be explained by delayed initiation of rehabilitation after TBI. A longer PTA duration generally reflects more extensive injury to memory and attention networks [43]. If rehabilitation begins late, functional recovery can be further limited. Eastvold et al. (2013) reported that patients who started rehabilitation earlier were more likely to return to work at one year [44], which supports the importance of timely intervention in this group. However, it is also important to acknowledge that clinically, ALOS is affected by multiple factors and may not be so easily modifiable. This is especially so, since the cause of prolonged ALOS might be due to the nature of the TBI itself—severe TBI patients are more likely to have complications requiring longer stay in the acute hospital to recover.

### 4.3. PTA Duration, Emergence and Functional Outcomes

Emergence from PTA signals the return of stable memory and orientation. Patients typically show clearer thinking and better day-to-day recall after this point [45]. In our study, age and longer initial PTA duration lowered the chance of emergence, while better cognitive FIM scores increased it.

Consequently, the functional outcomes of patients with prolonged PTA at admission and discharge were significantly lower compared to patients in PTA duration ≤ 30 days. Those with longer PTA durations had significantly lower admission and discharge FIM compared with shorter PTA ≤ 30 days (total admission FIM 48 vs. 68, total discharge FIM 83 vs. 102.5). Patients in PTA generally require more assistance for self-care tasks and suffer from cognitive impairments [46]. These characteristics of PTA contribute to lower motor and cognitive FIM scores. This study demonstrated that every score lost in the total admission FIM from 40 and below increased the odds of prolonged PTA by 3.35 times.

This study also showed that a higher proportion of patients in prolonged PTA were institutionalized post-discharge (26.7% vs. 6.8%). This is despite a third of the patients in the prolonged PTA subgroup managing to achieve a total discharge FIM score of 91 and above. There might be a few possible reasons to explain this. A high FIM score may not reflect all abilities needed for safe independent living. Patients with TBI can have persistent issues with judgement, behaviour, or executive functioning that are not captured well by FIM subscores [47]. Social and family factors may also influence the discharge destination. In Singapore, smaller households, dual-income families, and fewer available caregivers can make home discharge difficult [48,49]. Social isolation, particularly among older adults, may further increase the likelihood of institutionalization [50].

A multicentre study on risk factors for institutionalization after TBI inpatient rehabilitation cited prolonged PTA, older age, and living alone as risk factors [51]. This is similar to our study population. In the long term, prolonged PTA leads to increase long-term socioeconomic implications like work instability, divorce, and reduced chances of higher education [52]. Data on premorbid living situation or caregiver availability was unavailable in this study. It will be useful for future studies to incorporate social variables, which may be critical for discharge planning in older patients with prolonged PTA.

#### Study Limitations

Limitations of this study include missing data due to its retrospective nature. Furthermore, the data collected originated exclusively from one centre and had a limited sample size, which could reduce the applicability of the results to other populations. There might be potential selection bias as these are patients were admitted to a tertiary neurorehabilitation unit with sufficient data to analyse PTA and FIM, which is not necessarily representative of all TBI survivors, especially the very mild and very severe, who may bypass such services.

Continuous data was presented as binary outcomes which could lead to loss of some information.

We acknowledge that the regression models are exploratory and should be validated in larger or multicentre cohorts.

The study dataset also lacked data in relation to neuroimaging correlates, cognitive, vocational, and psychiatric or quality of life outcomes. Future research with larger sample sizes may address this limitation.

As per our local institution practice., WMPTAS is used for most patients while O-Log is used as the assessment tool in the oldest and/or premorbidly impaired. Although this practice is pragmatic and reflects the reality of the patient cohort, we acknowledge that there might be some heterogeneity in how PTA duration is measured.

## 5. Conclusions

This study demonstrated that older age at adult TBI onset and longer ALOS significantly increased the risk of prolonged PTA duration. Conversely, higher admission FIM score, lower age at admission, and shorter ALOS were associated with lower PTA duration. It might be possible that age may be acting as a surrogate for a cluster of vulnerability factors, including comorbidities, frailty, and baseline cognitive reserve. The interaction between age, comorbidity, and frailty may be an important area for future work.

Study findings support earlier transfer to rehabilitation milieu. This may be achieved by integration of a multidisciplinary team from day of admission to guide the rehabilitation process, prognostication, and facilitation of transition to a rehabilitation unit. Patients who require prolonged acute care due to various reasons like medical or surgical complications, etc., should continue receiving access to early mobilization and bedside interventions such as arousal optimization, daily reorientation, and orthostatic exercises. This will optimize their rehabilitation admission FIM scores and reduce the risk of prolonged PTA.

## Figures and Tables

**Table 1:** (a) Univariate analysis of demographic independent factors between PTA duration ≤30 days and >30 days (n = 189). (b) Univariate analysis of acute TBI factors between PTA duration ≤30 days and >30 days (n = 189).

Variable	Total (n = 189)	PTA Duration ≤ 30 Days (n = 88)	PTA Duration > 30 Days (n = 101)	*p*-Value
(a)				
Age				
Age (years), median (IQR)	64.0 (26.0)	59.5 (27.7)	66.0 (23.0)	0.017 ^a^
Age < 55 years, n (%)	67 (35.4%)	39 (44.3%)	28 (27.7%)	0.017 ^b^
Age ≥ 55 years, n (%)	122 (64.6%)	49 (55.7%)	73 (72.3%)	
Sex, n (%)				
Male	145 (76.7%)	69 (78.4%)	76 (75.2%)	0.608 ^b^
Female	44 (23.3%)	19 (21.6%)	25 (24.8%)	
Race, n (%)				
Chinese	148 (78.3%)	65 (73.9%)	83 (82.2%)	0.167 ^b^
Non-Chinese	41 (21.7%)	23 (26.1%)	18 (17.8%)	
Employed, n (%)				
Yes	116 (61.4%)	60 (68.2%)	56 (55.4%)	0.073 ^b^
CCI, median (IQR)	2.0 (4.0)	2.0 (4.0)	3.0 (3.0)	0.055 ^a^
Presence of at least 1 comorbidity, n (%)				
Hypertension	68 (36%)	29 (33%)	39 (38.6%)	0.419 ^b^
Hyperlipidaemia	69 (36.5%)	31 (35.2%)	38 (37.6%)	0.733 ^b^
Diabetes mellitus	46 (24.3%)	19 (21.6%)	27 (26.7%)	0.411 ^b^
Chronic smoker	23 (12.2%)	12 (13.6%)	11 (10.9%)	0.565 ^b^
Chronic alcohol drinker	17 (9%)	7 (8%)	10 (9.9%)	0.641 ^b^
(b)				
Aetiology of TBI				
Road traffic accident	74 (39.2%)	35 (39.8%)	39 (38.6%)	0.829 ^b^
Fall	104 (55%)	47 (53.4%)	57 (56.4%)	
Others	11 (5.8%)	6 (6.8%)	5 (5%)	
Admission GCS severity, n = 184 * (%)				
Mild (13–15)	105 (55.6%)	52 (59.1%)	53 (52.5%)	0.333 ^b^
Moderate (9–12)	45 (23.8%)	22 (25%)	23 (22.8%)	
Severe (3–8)	34 (18%)	12 (13.6%)	22 (21.8%)	
ICU admission, n = 186 * (%)				
Present	130 (68.8%)	54 (61.4%)	76 (75.2%)	0.029 ^b^
ICU LOS (days), median (IQR)	4.0 (4.5)	3.0 (3.0)	5.0 (6.2)	0.001 ^a^
Neurosurgical intervention, n (%)				
Performed	90 (47.6%)	36 (40.9%)	54 (53.5%)	0.085 ^b^
EVD	9 (4.8%)	0 (0%)	9 (8.9%)	0.004 ^b^
ICP monitor	43 (22.8%)	15 (17%)	28 (27.7%)	0.081 ^b^
Craniotomy	33 (17.5%)	13 (14.8%)	20 (19.8%)	0.364 ^b^
Decompressive craniectomy	23 (12.2%)	10 (11.4%)	13 (12.9%)	0.752 ^b^
Clot evacuation	38 (20.1%)	16 (18.2%)	22 (21.8%)	0.538 ^b^
Tracheostomy	4 (2.1%)	0 (0%)	4 (4%)	0.059 ^b^
VP shunt	2 (1.1%)	0 (0%)	2 (2%)	0.184 ^b^
Skull fracture, n = 127 * (%)				
Present	70 (37%)	37 (42%)	33 (32.7%)	0.313 ^b^
Acute TBI seizures, n (%)				
Present	22 (11.6%)	8 (9.1%)	14 (13.9%)	0.308 ^b^
Acute LOS (days), median (IQR)	18.0 (14.0)	14.0 (10.0)	23.0 (17.0)	<0.001 ^a^
Rehab LOS (days), median (IQR)	28.0 (21.0)	21.0 (14.0)	34.0 (22.0)	<0.001 ^a^
PTA emergence, n (%)				
Emergence	154 (81.5%)	77 (87.5%)	77 (76.2%)	0.047 ^b^
Complications during rehab stay, n (%)				
Present	97 (51.3%)	39 (44.3%)	58 (57.4%)	0.072 ^b^
Types of complication				
Medical	96 (50.8%)	42 (47.7%)	54 (53.5%)	0.431 ^b^
Neurosurgical	18 (9.5%)	5 (5.7%)	13 (12.9%)	0.093 ^b^
ACUR and reasons, n (%)				
Present	22 (11.6%)	7 (8.0%)	15 (14.9%)	0.140 ^b^
Neurosurgical causes	13 (6.9%)	5 (5.7%)	8 (7.9%)	0.544 ^b^
Medical causes	12 (6.3%)	3 (3.4%)	9 (8.9%)	0.122 ^b^
FIM (admission)				
Total FIM, median (IQR)	61.0 (38.0)	68.0 (28.2)	48.0 (37.0)	<0.001 ^a^
Motor FIM, median (IQR)	40.0 (29.0)	44.5 (23.0)	32.0 (26.0)	<0.001 ^a^
Cognitive FIM, median (IQR)	18.0 (14.0)	21.0 (12.2)	16.0 (15.0)	0.001 ^a^
FIM (Discharge)				
Total FIM, median (IQR)	92.0 (15.0)	102.5 (20.4)	83.0 (28.0)	<0.001 ^a^
Motor FIM, median (IQR)	67.0 (34.0)	74.5 (17.0)	58.0 (24.0)	<0.001 ^a^
Cognitive FIM, median (IQR)	26.0 (9.0)	29.0 (6.0)	23.0 (8.0)	<0.001 ^a^
Calculated FIM scores				
Td-FIM > 90, n (%)	102 (54.0%)	68 (77.3%)	34 (33.7%)	<0.001 ^b^
Td-FIM ≤ 90, n (%)	87 (46.0%)	20 (22.7%)	67 (66.3%)	
Discharge destination, n (%)				
Home	156 (82.5%)	82 (93.2%)	74 (73.3%)	<0.001 ^b^
Institution	33 (17.5%)	6 (6.8%)	27 (26.7%)	
Carer needed, n = 188 * (%)				
Yes	152 (80.4%)	64 (72.7%)	88 (87.1%)	0.018 ^b^

^a^ Mann–Whitney-U test; ^b^ Pearson Chi-Square test. * Missing data. N = 189 unless otherwise stated. Legends: PTA—post-traumatic amnesia, IQR—interquartile range, CCI—Charlson Comorbidity Index, TBI—traumatic brain injury, GCS—Glasgow Coma Scale, ICU—Intensive Care Unit, LOS—length of stay, FIM—Functional Independence Measure, Td-FIM—Total discharge Functional Independence Measure, ACUR—Acute Care Unit Return.

**Table 2:** Binary logistic regression analysis of factors associated with prolonged PTA (>30 days). Accuracy 0.702.

Variables	B	Exp (B)	95% CI	*p*-Value
Age ≥ 55 (1 = Yes)	1.723	5.602	1.473, 21.30	0.011
Presence of Motor Impairment (1 = Yes)	0.376	1.457	0.588, 3.608	0.416
ALOS (Days)	0.141	1.152	1.083, 1.225	<0.001
Charlson CCI	0.117	1.124	0.821, 1.539	0.466
ICU Admission (1 = Yes)	0.076	1.079	0.424, 2.747	0.873
Decompressive Craniectomy	0.358	1.431	0.419, 4.884	0.567
Ta-FIM (1 = Ta-FIM ≤ 40)	1.209	3.350	1.280, 8.768	0.014

Legends: ALOS—acute length of stay, CCI—Charlson Comorbidity Index, ICU—Intensive Care Unit, LOS—length of stay, FIM—Functional Independence Measure, Ta-FIM—Total admission Functional Independence Measure, PTA—post-traumatic amnesia.

**Table 3:** Logistic regression model for post-traumatic amnesia emergence. Accuracy: 0.84.

Variables	B	Exp (B)	95% CI	*p*-Value
PTA Duration (Days)	−0.072	0.930	0.893, 0.969	<0.001
Age ≥ 55 (1 = Yes)	−1.828	0.161	0.03, 0.847	0.031
ALOS (Days)	0.109	1.116	1.024, 1.216	0.012
Admission FIM Motor	−0.044	0.957	0.912, 1.004	0.073
Admission FIM Cognitive	0.141	1.151	1.038, 1.276	0.007
Neurosurgical Complications	−1.656	0.191	0.036, 1.003	0.051

Legends: ALOS—acute length of stay, FIM—Functional Independence Measure, PTA—post-traumatic amnesia.

## Data Availability

The data presented in this study are available on request from the corresponding author due to ethical reasons.

## Supplementary Materials

The following supporting information can be downloaded at: https://www.mdpi.com/article/10.3390/life16020203/s1, S1: Ethics Approval, S2: STROBE Statement, S3: Figure showing relationship between PTA duration and Clinical Variables.
