# Supplementary figures and images for: Factors Affecting Recovery from Post-Traumatic Amnesia During Inpatient Brain Injury Rehabilitation: A Retrospective Cohort Study
Source: Life (Basel). 2026 Jan 26;16(2):203. doi: 10.3390/life16020203 (PMC12941990; doi:10.3390/life16020203)

Relationship between PTA Duration and Clinical Variables

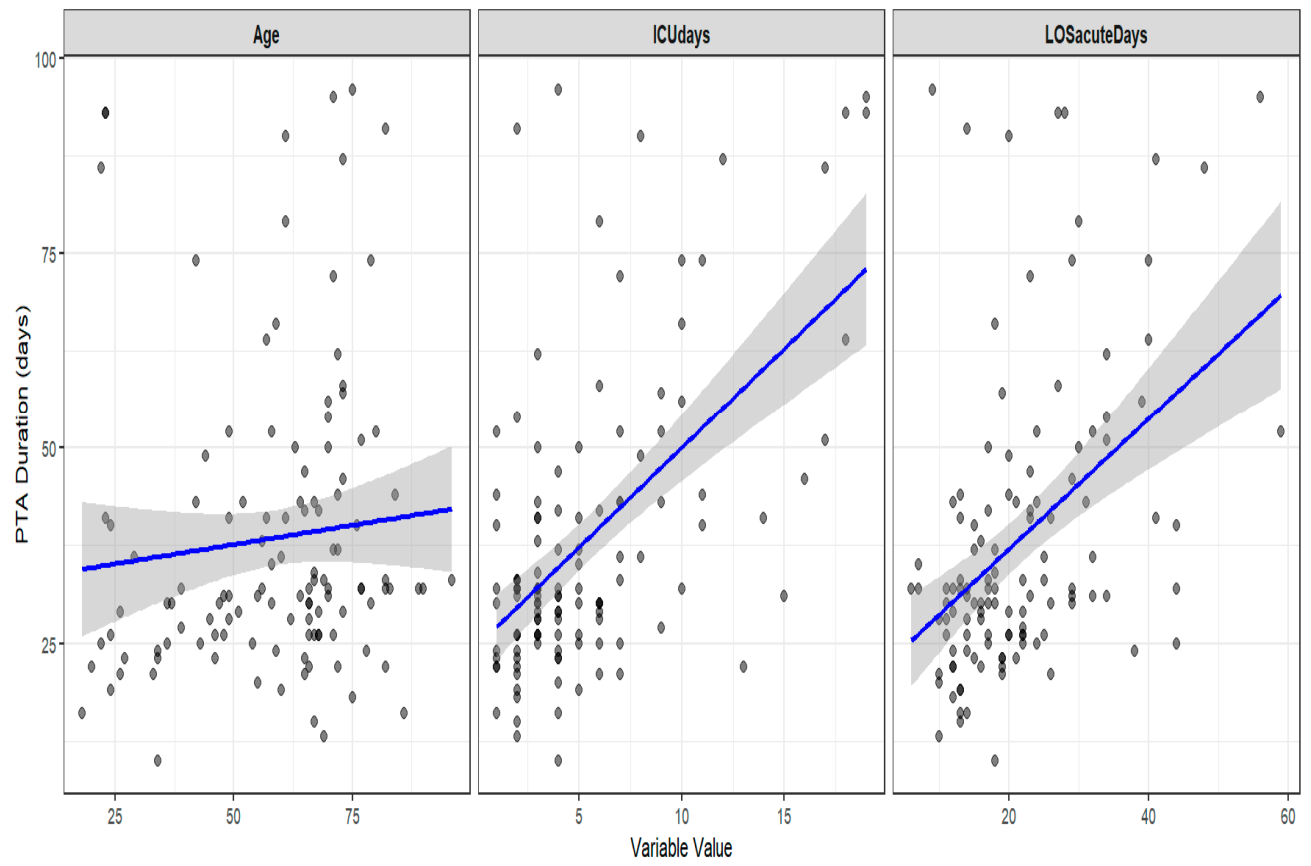

Supplement: Supplementary file 1 [file life-16-00203-s001.zip › S3. Relationship between PTA duration and Clinical Variables Graph.pdf]
