# Supplementary material for: Factors Affecting Recovery from Post-Traumatic Amnesia During Inpatient Brain Injury Rehabilitation: A Retrospective Cohort Study
Source: Life (Basel). 2026 Jan 26;16(2):203. doi: 10.3390/life16020203 (PMC12941990; doi:10.3390/life16020203)
Supplement: Supplementary file 1 [file S1. 2023_00173 08-06-2023.pdf › S1. 2023_00173 08-06-2023.pdf]

NHG DSRB Ref: **2023/00173**

08 June 2023

Dr Llewelyn Tan
Department of Rehabilitation Medicine
Tan Tock Seng Hospital

Dear Dr Tan

NHG DOMAIN SPECIFIC REVIEW BOARD (DSRB) APPROVAL

STUDY TITLE: Investigating the impact of post traumatic amnesia(PTA) metrics on functional outcome during inpatient rehabilitation for traumatic brain injury(TBI): A retrospective study.

We are pleased to inform you that the NHG Domain Specific Review Board has approved the application as titled above to be conducted in **Tan Tock Seng Hospital**.

The approval period is from **08 June 2023** to **07 June 2024**. The NHG DSRB reference number for this study is **2023/00173**. Please use this reference number for all future correspondence.

In accordance with the NHG Data Access & Sharing Policy, NHG investigators and study team members who require access to data that is not collected or created directly by the research team from research subjects or research materials (e.g. through interviews, surveys or experiments) should consult the relevant Data Exchange Office (DXO) to ascertain if a data access request should be raised. The NHG Institutional and Cluster DXO's details can be found at https://mynhg.nhg.com.sg/clst/nhgdxo/Pages/default.aspx.

The documents reviewed are:

a) NHG DSRB Application Form: **Version No. 1**
b) List of Variables: **Version 3 dated 18 May 2023**

The NHG DSRB has approved your request for waiver of informed consent.

Continued approval is conditional upon your compliance with the following requirements:

1. Translated Informed Consent Forms (fully translated and short consent forms) and other translated documents are not required to be submitted to DSRB. It is the

responsibility of the Principal Investigator to ensure that the translations for any document are an accurate reflection of the original approved content and to maintain the certification/documentation of the translations.

2. No deviation from or changes to the study should be implemented without documented approval from the NHG DSRB, except where necessary to eliminate apparent immediate hazard(s) to the study subjects.

3. Any deviation from or changes to the study to eliminate an immediate hazard should be promptly reported to the NHG DSRB within seven calendar days.

4. Principal Investigators and study team members are responsible for adhering to institutional guidelines, where applicable.

5. Please note that for studies requiring CTA/CTN/CTC, apart from the approval from NHG DSRB, no deviation from, or changes of the Research Protocol and Informed Consent Form should be implemented without documented approval from the Health Sciences Authority unless otherwise advised by the Health Sciences Authority.

6. Please submit the following to the NHG DSRB:

   a. All Unanticipated Problems Involving Risk To Subjects Or Others (UPIRTSOs) must be reported to the NHG DSRB. For more than minimal risk studies, all problems involving local deaths **must be reported as soon as possible**, but not later than 7 calendar days after first knowledge by the Investigator, regardless of the causality and expectedness of the death event, and any additional relevant information about the death should be reported within 8 calendar days of making the initial report. For no more than minimal risk studies, only problems involving local deaths that are related or possibly related to the study **must be reported as soon as possible**, but not later than 7 calendar days after first knowledge by the Investigator, and any additional relevant information about the death should be reported within 8 calendar days of making the initial report. For problems which are life threatening, it **must be reported as soon as possible**, but not later than 7 calendar days after first knowledge by the investigator, and any additional relevant information about the problems should be reported within 8 calendar days of making the initial report. All other problems that fulfil the UPIRTSOs reporting criteria **must be reported as soon as possible** but not later than 15 calendar days after first knowledge by the Investigator.

   b. Report(s) on any new information that may adversely affect the safety of the subject or the conduct of the study.

   c. NHG DSRB Study Status Report Form – this is to be submitted 4 to 6 weeks prior to expiry of the approval period. The study cannot continue beyond **07 June 2024** until approval is renewed by the NHG DSRB.

d. Study completion – this is to be submitted using the NHG DSRB Study Status Report Form within 4 to 6 weeks of study completion.

With the enactment of the Human Biomedical Research Act, Health Products Act, Medicines Act and their subsidiary legislations, Principal Investigators are reminded to ensure that their research complies with the regulatory requirements stipulated in the applicable Acts. Contraventions under any of these Acts are criminal offences and would result in fines or imprisonment or both, subject to the nature of the offence.

Established since May 2006, the NHG Research Quality Management (RQM) Programme seeks to promote the responsible conduct of research in a research culture with high ethical standards, identify potential systemic weaknesses and make recommendations for continual improvement. Hence, this research study may be randomly selected for a review by the Research Quality Management (RQM) team. For more information, please visit www.research.nhg.com.sg.

The NHG DSRB operates in accordance to the ICH GCP and all applicable laws and regulations.

Yours Sincerely

A/Prof Chan Mun Yew Patrick
Chairman
NHG Domain Specific Review Board D

Cc: Institutional Representative, TTSH
c/o Clinical Research & Innovation Office, TTSH
Departmental Representative of Rehabilitation Medicine, TTSH

(This is an electronic-generated letter. No signature is required.)
