# Supplementary material for: Factors Affecting Recovery from Post-Traumatic Amnesia During Inpatient Brain Injury Rehabilitation: A Retrospective Cohort Study
Source: Life (Basel). 2026 Jan 26;16(2):203. doi: 10.3390/life16020203 (PMC12941990; doi:10.3390/life16020203)
Supplement: Supplementary file 1 [file S1. 2023_00173_Extension_14-03-2024.pdf › S1. 2023_00173_Extension_14-03-2024.pdf]

NHG DSRB Ref: 2023/00173

14-Mar-2024

Dr Llewelyn Tan
Department of Rehabilitation Medicine
Tan Tock Seng Hospital

Dear Dr Llewelyn Tan

SIX (6) MONTHS EXTENSION OF NHG DOMAIN SPECIFIC REVIEW BOARD (DSRB) APPROVAL

Study Title:Investigating the impact of post traumatic amnesia(PTA) metrics on functional outcome during inpatient rehabilitation for traumatic brain injury(TBI): A retrospective study.

We are pleased to inform you that the NHG DSRB has extended the approval period for the application as titled above, by six (6) months from the study's last expiry date. The approval has been extended to **07-Dec-2024**

For cross-cluster studies, this approval is mutually recognised by SingHealth Centralised Institutional Review Board (CIRB).

Continued approval is conditional upon your compliance with the following requirements:

1. Only the approved Informed Consent Form should be used, if applicable. It must be signed by each subject prior to initiation of any protocol procedures. In addition, each subject should be given a copy of the signed consent form.

2. Translated Informed Consent Forms (fully translated and short consent forms) and other translated documents are not required to be submitted to DSRB. It is the responsibility of the Principal Investigator to ensure that the translations for any document are an accurate reflection of the original approved content and to maintain the certification/documentation of the translations.

3. No deviation from, or changes of the protocol should be implemented without documented approval from the NHG DSRB, except where necessary to eliminate apparent immediate hazard(s) to the study subjects.

4. Any deviation from, or a change of, the protocol to eliminate an immediate hazard should be promptly reported to the NHG DSRB within seven calendar days.

5. Principal Investigators and study team members are responsible for adhering to institutional guidelines, where applicable.

6. Please note that for studies requiring CTA/CTN/CTC, apart from the approval from NHG DSRB, no deviation from, or changes of the Research Protocol and Informed Consent Form should be implemented without documented approval from the Health Sciences Authority unless otherwise advised by the Health Sciences Authority.

National Healthcare Group
Adding years of healthy life

7. Please submit the following to the NHG DSRB:

   a. All Unanticipated Problems Involving Risk To Subjects Or Others (UPIRTSOs) must be reported to the NHG DSRB. For more than minimal risk studies, all problems involving local deaths **must be reported as soon as possible**, but not later than 7 calendar days after first knowledge by the Investigator, regardless of the causality and expectedness of the death event, and any additional relevant information about the death should be reported within 8 calendar days of making the initial report. For no more than minimal risk studies, only problems involving local deaths that are related or possibly related to the study **must be reported as soon as possible**, but not later than 7 calendar days after first knowledge by the Investigator, and any additional relevant information about the death should be reported within 8 calendar days of making the initial report. For problems which are life threatening, it **must be reported as soon as possible**, but not later than 7 calendar days after first knowledge by the investigator, and any additional relevant information about the problems should be reported within 8 calendar days of making the initial report. All other problems that fulfil the UPIRTSOs reporting criteria **must be reported as soon as possible** but not later than 15 calendar days after first knowledge by the Investigator.

   b. Report(s) on any new information that may adversely affect the safety of the subject or the conduct of the study.

   c. NHG DSRB Study Status Report Form – this is to be submitted 4 to 6 weeks prior to expiry of the approval period. The study cannot continue beyond **07-Dec-2024** until approval is renewed by the NHG DSRB.

   d. Study completion – this is to be submitted using the NHG DSRB Study Status Report Form within 4 to 6 weeks of study completion or termination.

Established since May 2006, the NHG Research Quality Management (RQM) Program seeks to promote the responsible conduct of research in a research culture with high ethical standards, identify potential systemic weaknesses and make recommendations for continual improvement. Hence, this research study may be randomly selected for a review by the Research Quality Management (RQM) team. For more information, please visit www.research.nhg.com.sg.

The NHG DSRB operates in accordance to the ICH GCP, and all applicable laws and regulations.

Yours Sincerely

NHG Domain Specific Review Board D1

CC: Institutional Representative, Tan Tock Seng Hospital

Departmental Representative of Tan Tock Seng Hospital, Rehabilitation Medicine

(This is an electronic-generated letter. No signature is required.)
